# 

**Published:** 1867-01

**Authors:** 


### THE CHICAGO MEDICAL JOURNAL.

**E. L. HOLMES, M.D., H. M. LYMAN, M.D., R. M. LACKEY, M.D., Editors.**

All letters for the JOURNAL should be addressed to R. M. LACKEY, M.D., P.O. Box 2175, Chicago, Ill.

**TERMS---$2 PER ANNUM, IN ADVANCE.**

$3 if not paid before 1st of July in each year. Single copies, 25 cents.

BOOKS AND PAMPHLETS RECEIVED.

Opinion Relative to the Testamentary Capacity of the late James C. Johnston. By Wm. A. Hammond, M.D., etc.

Transactions of the Medical Society of the State of Pennsylvania, at its Seventeenth Annual Session.

Report on the Sanitary Relations of the State of Kansas. By C. A. Logan, M.D.

Report on the Climatology of Kansas. By Tiffin Sinks, M.D. A valuable contribution to our meteorological records.

Infantile Paralysis and its Attendant Deformities. By Charles F. Taylor, M.D. Philadelphia: J. B. Lippincott & Co. pp. 119.

For sale by S. C. Griggs & Co., 41 Lake St., Chicago.

The Message and Documents, for 1865–6. From J. H. Baxter, Surgeon, U.S. Vols., Washington D.C.

---

We have received the annual report of the Surgeon-General of the U.S. Army, for the year ending July 1st, 1866—a concise and interesting pamphlet of eight pages. After mentioning the fact of the reduction of the army to a peace footing, the author says:—

The comfort and proper medical treatment of the sick and wounded are now secured in well arranged Post Hospitals, of which there are at present one hundred and eighty-seven (187) with a total capacity of ten thousand eight hundred and eighty-one (10,881) beds. All perishable articles of medicines and hospital supplies in excess of the requirements of a peace establishment have been disposed of by public sale at advantageous rates, and the reserved supplies concentrated at the Purveying Depots in New York, Philadelphia, Washington, St. Louis, New Orleans, and San Francisco.

Great progress has been made in furnishing artificial limbs to maimed soldiers:—

From date of Act of Congress, (July 16, 1862,) authorizing artificial limbs to be furnished ,to July 1, 1866, there have bend supplied by this Department, to maimed soldiers, three thousand nine-hundred and eighty-one (3,981) legs; two thousand two hundred and forty (2,240) arms; nine (9) feet; fifty-five (55) hands; one hundred and twenty-five (125) surgical apparatus, and it is estimated that not more than one thousand (1,000) limbs remain still to be supplied at a cost probably of seventy thousand dollars ($70,000). Should the appropriations for this purpose be continued it is recommended that upon furnishing the evidence now required to obtain an artificial limb, the applicant receive, from a Medical Disbursing Officer, under such regulations as may be prescribed by the Secretary of War, its present established money value in lieu of an order upon a manufacturer. Such an arrangement would include those cases in which from the nature of the injury and operation, no limb or (surgical) appliance can be advantageously adopted, by extending to them the same allowance now made to their more fortunate fellow-sufferers.

We are informed that:—

The average mean strength of white troops for the year as reported, was one hundred thousand one hundred and thirty-three (100,133), and the proportion of deaths, from all causes, to cases treated was one to every fifty-two.

The report of colored troops represents the average mean strength for the same period as fifty-three thousand five hundred and forty-one (53,541), among whom the proportion of cases taken sick was greater than with the white troops, and the mortality rate, one death to every twenty-nine cases treated.

There were remaining in General Hospitals, June 30, 1865, and admitted during the year, sixty-four thousand four hundred and thirty-eight (64,438) patients, of whom, on the 30th June, 1866, only ninety-seven (97) remained under treatment.

We are glad to learn that:—

The preparation for publication of the Medical and Surgical History of the War has been prosecuted with energy, much of the manuscript and several of the illustrations for the first volume being completed. The Army Medical Museum continues to increase in value and usefulness, and the greater security and additional accommodations of the building to which it will be shortly removed admits of the addition of a great number of interesting and instructive specimens not hitherto available for want of space.

The number of casualties from the commencement of the war to July 1st, 1866, in the Regular and Volunteer Medical Staff, was three hundred and thirty-six, *viz.:*—

Killed in battle, twenty-nine (29); killed by accident, twelve (12); died of wounds, ten (10); died in rebel prison, four (4); died of yellow fever, seven (7); died of Cholera, three (3); died of other diseases, two hundred and seventy-one (271); making a total of three hundred and thirty-six (336).

During the war thirty-five (35) Medical Officers were wounded in battle.

That the Medical Department of the Army will be adequately represented in the great Paris Exposition, is rendered certain by the following paragraph:—

The improvements in Hospital construction and equipment, in surgical appliances, in means of transportation of sick and wounded, etc., resulting from the vast experience of the War, are considered worthy of exhibition as an evidence of National progress, and with this view, models of U. S. General Hospitals, with their equipment, of ambulances, litters, medicine wagons, etc., have been prepared, and will be forwarded through the proper channels as the contribution of the Medical Department, U. S. A., to the Paris Exposition.

To the report is appended a statement of the quantity of of medical supplies issued during the war, from the purveying depots at New York City, Philadelphia, Baltimore, Washington, Cincinnati, Louisville, and St. Louis. The figures are simply enormous. We give a few specimens, regretting that our space will not permit the reprint of the whole list as a contribution to the curiosities of medical literature. The quantity of ether was not large, only 987,687 oz. were issued. Chloroform seems to have been the favorite anæsthetic, 1,588,066 oz. having been consumed. The spirits of nitric ether was in great demand, no less than 1,688,943 oz. having been required. Aconite was the favorite arterial sedative, if we may judge from the fact that 202,842 oz. of its fluid extract were consumed, against 48,759 oz. of the fluid extract of veratrum viride. Sulphate of magnesia found favor to the extent of 537,712 ℔s. But this was by no means the *royal cathartic:* defecation was procured by the action of 84,767 oz. of aloes,

127,027 oz. of calomel, 313,647 oz. of blue mass, 220,076 qts. of castor-oil, 28,486 oz. of croton oil, 141,875 oz. of rhubarb, and 655,982 dozen of compound cathartic pills. The consumption of opium and its various compounds was correspondingly great, *e.g.*:—448,864 oz. of opium, and 901,467 oz. of its tincture. Of quinine, 723,521 oz. were used. These are magnificent items, but it is only when one approaches the subject of alcoholic stimulants, that the exigencies of the occasion become fully apparent. 590,604 quarts of alcohol, 562,221 quarts of brandy, 913,729 quarts of wine, 1,113,690 quarts of porter, and 2,420,785 quarts of whiskey barely sufficed to sustain the fainting energies of our sick and wounded heroes. No wonder that Mosby, and Morgan, and Forrest were so eager to intercept our hospital trains!

Several of the items look queerly out of place in a list of army stores; for instance:—three hundred and fifteen obstetrical instrument cases, flanked by five hundred and thirty-seven copies of brother Bedford's Midwifery. Four hundred and twenty-six speculums, with seven hundred and thirty-seven vaginal syringes, were actively used during the war. When the volumes of the Army Medical Report are published, we shall hope to learn how often the operation for the relief of vesico-vaginal fistula was performed under fire, in Virginia. But these seem insignificant trifles when we turn to the masculine side of the question:—154,154 little syringes were ordered, for the comfort of the gentlemen who consumed 1,292,129 oz. of copaiba. Thus was the ancient reputation of Mars once more exalted, while the parturient pangs of Venus were gently urged with 22,471 oz. of ergot. Verily, it was the greatest war the century has seen.

---

The *Atlantic Monthly* enters on its nineteenth volume with an array of distinguished names and sterling articles that promise well for the coming year. The January number contains the first instalment of Dr. Holmes' story, "The Guardian Angel," in which will be found the same old charm that so fascinated the readers of the *Autocrat*, the *Professor*, and *Elsie*

*Venner;* a humorous story in verse, by James Russell Lowell; a graphic sketch of Henry Ward Beecher's church, with some pertinent reflections upon modern church-going, by James Parton; a legend in verse, told only as Whittier can tell it; a poem entitled "Terminus," (on Growing Old,) by R. W. Emerson; and a spirited translation of the contest between Achilles and Agamemnon, from the First Book of the Iliad, by W. C. Bryant.

---

We feel that we cannot confer upon our subscribers who are blessed with children a greater favor than by calling their attention to the new monthly, *The Riverside Magazine for Young People.* It is a double-columned quarto of 48 pages, illustrated with beautiful wood-cuts, and printed in that perfect style which has made the issues of the Riverside press so justly celebrated. The editorial department is in the hands of our old friend, Horace Scudder, an author whose reputation has already been established among the children, by a number of pleasing articles from his pen. The veteran writer of the *Rollo Books* has commenced a series of narratives, which will appear with every monthly issue; and, if we may venture to predict from the appearance of the first number, the new magazine will lead the way in the race of the juvenile monthlies. Hurd & Houghton, 459 Broome Street, New York, are the publishers. Price $2.50 a year, in advance.

---

We would refer our readers to the report on the milk of the Elgin Milk Condensing Company, as found in the proceedings of the Chicago Medical Society, page 23 of this journal.

---

THE Dissecting Room of Rush Medical College will be open during the months of February, March, and April, under the direction of Drs. POWELL and LACKEY. Every facility will be afforded students for the study of practical anatomy.

---

OUR thanks are due for the promptness with which our friends are sending in their subscriptions. With this number of the JOURNAL we return receipts for all payments received before the 12th of January. We trust that with our February issue we may be able to forward a receipt for 1867 to each one of our numerous readers.

---

REQUESTS for back numbers or extra copies of the JOURNAL should be forwarded by the middle of the month, in order to secure attention before the following month. Letters, however, which inclose postage stamps sufficient to prepay such matter as may be desired, will receive instant notice, and will receive a reply by the next post.

